# Suicide Gene-Engineered Stromal Cells Reveal a Dynamic Regulation of Cancer Metastasis

**DOI:** 10.1038/srep21239

**Published:** 2016-02-19

**Authors:** Keyue Shen, Samantha Luk, Jessica Elman, Ryan Murray, Shilpaa Mukundan, Biju Parekkadan

**Affiliations:** 1 Department of Surgery, Center for Engineering in Medicine and Surgical Services, Massachusetts General Hospital, Harvard Medical School and the Shriners Hospitals for Children, Boston, Massachusetts 02114, USA; 2 Harvard Stem Cell Institute, Cambridge, Massachusetts 02138, USA

## Abstract

Cancer-associated fibroblasts (CAFs) are a major cancer-promoting component in the tumor microenvironment (TME). The dynamic role of human CAFs in cancer progression has been ill-defined because human CAFs lack a unique marker needed for a cell-specific, promoter-driven knockout model. Here, we developed an engineered human CAF cell line with an inducible suicide gene to enable selective *in vivo* elimination of human CAFs at different stages of xenograft tumor development, effectively circumventing the challenge of targeting a cell-specific marker. Suicide-engineered CAFs were highly sensitive to apoptosis induction *in vitro* and *in vivo* by the addition of a simple small molecule inducer. Selection of timepoints for targeted CAF apoptosis *in vivo* during the progression of a human breast cancer xenograft model was guided by a bi-phasic host cytokine response that peaked at early timepoints after tumor implantation. Remarkably, we observed that the selective apoptosis of CAFs at these early timepoints did not affect primary tumor growth, but instead increased the presence of tumor-associated macrophages and the metastatic spread of breast cancer cells to the lung and bone. The study revealed a dynamic relationship between CAFs and cancer metastasis that has counter-intuitive ramifications for CAF-targeted therapy.

Host microenvironments can contribute to the growth, metastasis, and drug resistance of a tumor^1^. Many have begun to evaluate the cellular drivers of a tumor microenvironment (TME) for cancer therapy^2^. Yet, a TME is dynamic, with a changing landscape of stromal cell invasion from the periphery, cell differentiation, and apoptosis. From early carcinomas to late stage cancers, a multitude of stromal cell types are recruited to, activated and/or differentiated in the TME, including endothelial cells, fibroblasts, and various bone marrow-derived cells^3^. Temporal analysis of the cellular dynamics of a TME has been challenging, primarily because of the lack of unique markers to drive precise transgenic experiments that control the fate of stromal cells in a TME. A model that can specifically modify a stromal cell over time would enable an understanding of the exact roles of stromal cell types of interest in TME development and cancer progression.

Determining the contribution of a stromal cell in a TME also has therapeutic ramifications. A quintessential example of the challenge to study and modify TME cells can be found with cancer-associated fibroblasts (CAFs), a major cancer-promoting stromal component of the TME^4^. CAFs can produce paracrine growth factors to promote tumor growth, and proteolytic enzymes as well as secrete extracellular matrix to facilitate cancer cell migration and metastasis^5^. They can also communicate with other stromal cell types, for example, by recruiting endothelial progenitor cells to promote angiogenesis^6^, and/or promoting recruitment of monocytes to the tumor sites and their differentiation into pro-tumor M2 macrophages^7^. Experimental immunotherapies against CAF-expressing fibroblast activation protein (FAP) showed promising results in some pre-clinical models^8^,^9^,^10^. However, similar FAP-targeting therapies lacked clinical efficacy in human subjects^11^,^12^. Moreover, a T cell therapy against FAP-expressing cells in an animal model induced cachexia and lethal bone toxicity by unintentional targeting of FAP-expressing bone marrow stromal cells^13^. Optimal TME-targeted therapies demand an *in vivo* model that enables precise stromal cell elimination without prior knowledge of any stromal markers or “on target, off-tumor” effects.

This study directly addressed this challenge and established a model that enabled selective elimination of non-unique stromal cells in a human TME using a suicide gene engineering approach. CAF cells engineered with an inducible caspase gene were temporally killed during the progression of a human xenograft breast cancer model and multiple outcomes were monitored. The study revealed a dynamic relationship between CAFs in cancer metastasis that may contra-indicate CAF-targeted apoptotic therapies at early timepoints of tumor progression.

## Results and Discussion

### Apoptosis can be induced *in vitro* in transduced CAFs

**Figure 1:**
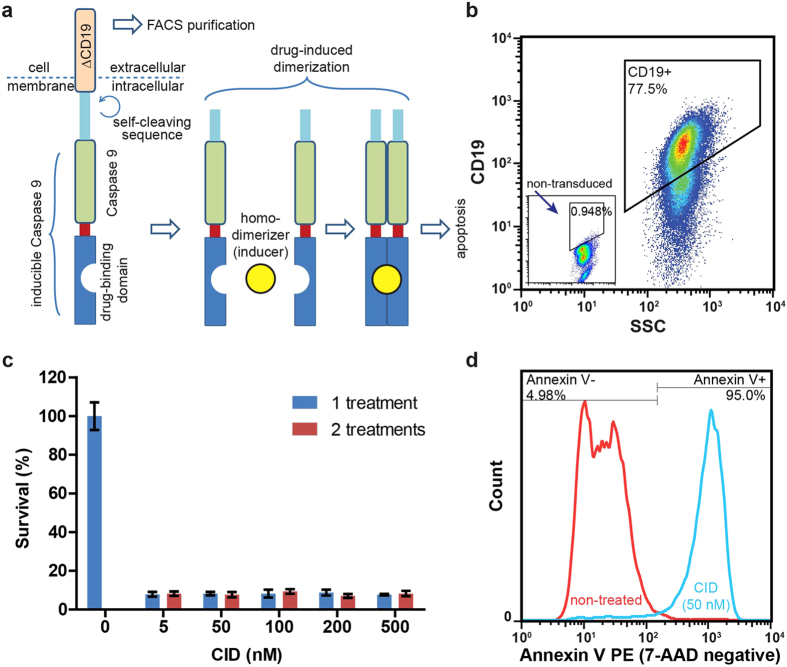
Establishment of suicide gene-engineered stromal cells. (**a**) The expressed protein from iCasp-ΔCD19 construct contains a CD19 cross-membrane domain for cell purification, and a self-cleavable inducible Caspase 9 (iCasp) domain. Dimerization of Caspase 9 will initiate cell apoptosis when the whole protein construct is dimerized by a homodimerizer at the drug-binding domain. (**b**) Flow cytometric analysis showed over 77.5% of the transduced CAF cells are positive for CD19 >5 passages after FACS purification. (**c**) The survival fraction of CAF-iCasp cells are uniformly low (<10%) across a wide range of CID homodimerizer treatment after one (24-hour) or two (48-hour) treatment doses. (**d**) Apoptosis analysis by Annexin V and 7-AAD co-staining shows over 95% of CAF-iCasp undergo apoptosis within 24  hours, after one dose of 50 nM CID treatment.

An inducible Caspase 9 construct (iCasp9-ΔCD19)^14^,^15^ was retrovirally introduced into a human CAF cell line^16^ to create CAF-iCasp cells (Fig. 1a). The expressed construct has a truncated CD19 extracellular and transmembrane domain (ΔCD19) for identification and purification by fluorescence-activated cell sorting (FACS) or other antibody-based methods. Flow cytometry analysis showed that 82 ± 4% (mean ± standard deviation, SD) of the cells were highly positive for CD19 (Fig. 1b), which was stable over 5 passages and was growth-competitive with uninfected cells. A self-cleaving sequence ensured separation between iCasp9 and ΔCD19 upon translation, and a drug-binding domain allows for binding/dimerization by a synthetic homodimerizer to trigger apoptosis through dimerized caspase 9 (Fig. 1a). When cells were exposed to a chemical inducer of dimerization (CID), AP20187, in a dose dilution study (from 5 nM to 500 nM) the survival of cells were uniformly <10% across the board (Fig. 1c) independent of exposure time to CID (24 or 48 hours). This response to CID suggested an “on/off” switch-like apoptotic behavior of the CAF-iCasp cells. The original non-transduced CAF cells did not respond to the CID drug. We further confirmed that over 95% of CAF-iCasp cells became apoptotic within 24 hours of CID treatment (Fig. 1d).

### Suicide gene-engineered CAF can be selectively eliminated *in vivo* in a xenograft model

**Figure 2:**
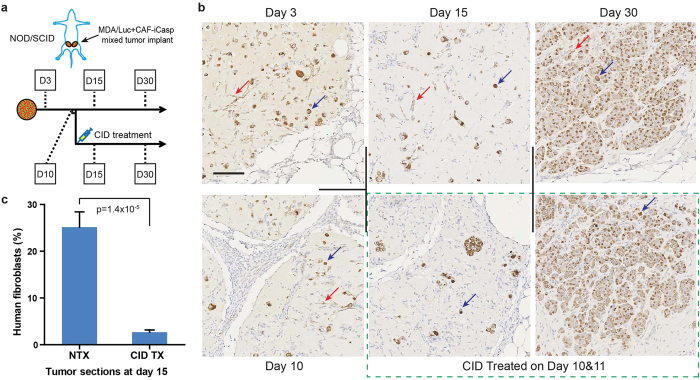
*In vivo* elimination of suicide gene-engineered stromal cells. (**a**) NOD/SCID mice were inoculated with MDA-Luc + CAF-iCasp tumors at mammary fatpads on both flanks, and half of the population was injected with CID homodimerizer at day 10 and 11. Animals were sacrificed on dash-line indicated days and tumor were stained with a human mitochondria-specific antibody. (**b**) Human mitochondria staining of xenograft tumor sections where MDA/Luc and CAF-iCasp cells were co-implanted into the mammary fatpads of NOD/Scid mice. Fibroblastic human CAF cells remain visible 30 days after implantation in the NTX condition, and hardly seen in the tumors from mice hosts treated with CID injection on day 10 and 11. Scale bar: 100 μm. (**c**) Morphology-based quantification shows dramatic reduction of human fibroblasts in tumors treated with CID drug (p = 1.4 × 10^−5^, Student’s t-test). Total images analyzed: NTX: n = 13; CID TX: n = 14. For *in vivo* study, N = 5 animals per endpoint, per condition. Error bars: standard error of the mean (SEM).

The engineered cell line was advanced to *in vivo* studies to confirm whether suicide-induction can be achieved *in situ* in a mouse model of cancer. We focused on breast cancer based on previous evidence that phenotypic changes in the fibroblastic stroma of breast cancer patients have been a strong predictor signature to poor outcomes^17^. MDA-MB-231 breast cancer cells carrying a luciferase gene^18^ (MDA/Luc) were co-implanted with equal numbers of CAF-iCasp cells into the mammary fat pads of immune compromised female NOD/SCID mice. A cohort of the animals were randomized and treated with two doses of CID drug on day 10 and 11 to eliminate CAF-iCasp cells. Tumor-bearing mice were sacrificed on day 3, 10, 15, and 30 to characterize tumor morphology and CAF distribution (Fig. 2a). Tumor sections were immunostained for human mitochondria to specifically detect the human origin MDA/Luc and CAF-iCasp cells. The two cell types were readily distinguishable by rounded epithelial morphology (blue arrows) and extended fibroblastic morphology (red arrows), respectively (Fig. 2b), in the tumors without CID treatment at all the time points in the 30-day study. We found implanted cells generally underwent an initial cell loss through day 10 and 15, with shrunk human cell areas in the tumor sections. By day 30, cancer cells had proliferated extensively to occupy the majority of the tumor sections; yet, distinct human CAF-iCasp cells were still visible as indicated by the red arrow. In contrast, human CAF-iCasp cells were largely absent from tumor sections at day 15 and 30 in the CID treated groups (Fig. 2b, green box; Fig. 2c), confirming CAF cells were selectively targeted and eliminated from the TME in our *in vivo* model.

### Intratumor cytokines have two distinct profiles in co-implanted tumors

**Figure 3:**
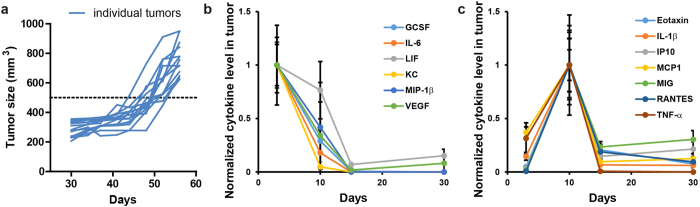
Tumor growth dynamics and intratumor cytokine response to CAF elimination. (**a**) In an 8-week *in vivo* study of MDA/Luc+CAF-iCasp tumor growth, tumor size does not demonstrate visible changes until 43~53 days into the experiment measured by an arbitrary threshold (500 mm^3^). (**b**) A collection of host (mouse) cytokines with peak level at day 3 followed by steady decline into day 30. (**c**) A collection of host cytokines with initial increase to peak level at day 10 followed by steady decline into day 30.

With confidence that we could specifically eliminate CAFs *in vivo*, the next objective was to determine when to eliminate CAF cells in the tumors in order to cause impactful changes in tumor development. We based this decision on the natural course of tumor dynamics. MDA/Luc were mixed with an equal number of CAF-iCasp cells and injected in the mammary fat pad of female NOD/SCID mice. Initial observation showed these co-implant xenograft tumors did not exhibit significant growth until 43~53 days into the experiment, with wide distribution of tumor sizes at any given time point during their exponential growth phase (Fig. 3a). On the other hand, morphological characterization (Fig. 2b) showed massive cancer cell growth (day 30) prior to apparent volumetric growth of tumor tissue. To better gauge tumor biology at a molecular and cellular level, we performed a multiplex cytokine analysis to determine the host (mouse) response to the co-implanted xenograft tumor on day 3, 10, 15, and 30. Surprisingly, we discovered two distinct classes of cytokine responses, namely a rapid response group (Fig. 3b) and a delayed response group (Fig. 3c). In the rapid response group, cytokines peaked on day 3, followed by a quick decline on day 10, and near-zero level on day 15 and 30. In the delayed response group, cytokines were initially relatively low, peaked on day 10, and then quickly declined by day 15. As most of these cytokines are associated with innate immunity, particularly macrophage functions, we hypothesized that day 3 and 10 represent critical turning points of the host immune reactions to the *in vivo* tumor development.

### Macrophage recruitment is enhanced in CAF-eliminated tumors

**Figure 4:**
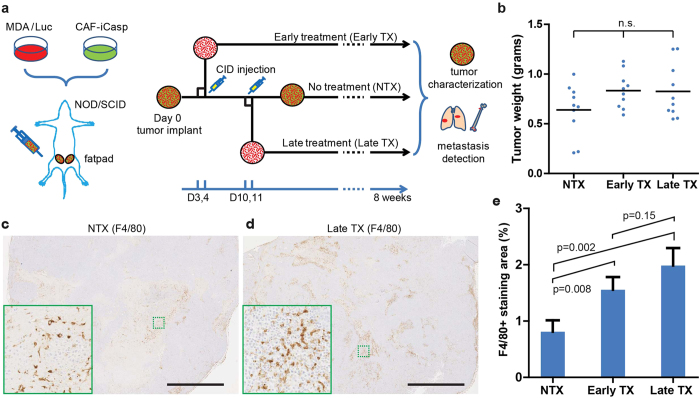
Three *in vivo* treatment conditions and phenotype analysis. (**a**) Design of three conditions for *in vivo* CAF elimination in xenograft model, and quantifiable read-outs. (**b**) Tumor sizes at the end of the study are not statistically different (p > 0.05, one-way analysis of variance (ANOVA)). F4/80 staining of (**c**) NTX tumors and (**d**) Late TX tumors at the end of the study. (**c**,**d**) Scale bar: 2 mm. Insets (solid green boxes) are magnified areas in the dashed green boxes. (**e**) Comparison of F4/80+ staining in the tumor tissue sections (Student’s t-test). Total images analyzed: NTX: n = 26; Early TX: n = 25; Late TX: n = 32. Statistical comparisons are Student’s t-test. For all experiments, N = 5 animals per condition. Error bars: SEM.

We then studied the impact of deleting CAF cells in the TME with respect to the progression of tumor growth, composition, and metastasis. Based on the intratumor cytokine dynamics, we chose day 3 (Early TX) and day 10 (Late TX) for the elimination of CAFs to cover these transitional stages. The animals were treated with CID drug through peritoneal injections on days 3 & 4 (Early TX) or 10 & 11 (Late TX) after tumor implantation, or without treatment (NTX), and tumors were monitored non-invasively until endpoint measurements were taken (Fig. 4a). We first measured whether CAF elimination at these time points had any impact on primary tumor growth and composition. Primary tumor weights in all the three conditions at the end of the 8-week study did not show significant difference (p > 0.05, one-way ANOVA) (Fig. 4b). We hypothesized that CAF elimination at these early time points could alter the macrophage populations in tumors (Fig. 3a,b). We next stained F4/80 in the tumor sections of the three treatment groups as an indicator of the involvement of macrophages in the tumor development. It was immediately clear by gross visual examination (Fig. 4c,d) that the tumors from NTX group had significantly (p < 0.05, Student’s t-test) less accumulation of F4/80 positive stain than those from both CID treated groups (Fig. 4e; also see Fig. 4c,d insets). A strong trend was observed between the Early TX and Late TX groups, with the Late TX tumors having a higher accumulation of F4/80 positive cells.

### Lung and bone metastases increase only in the late-treated animals

**Figure 5:**
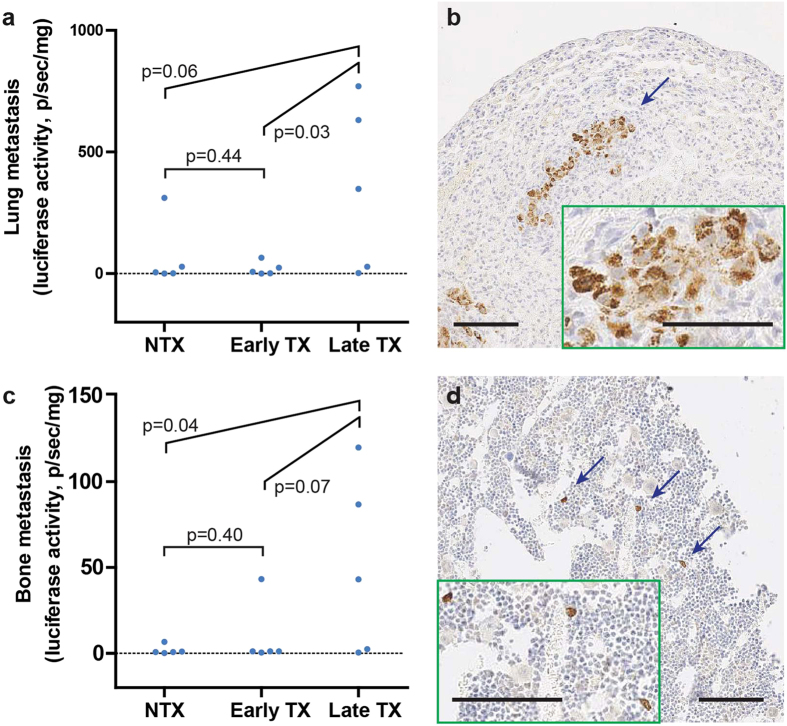
Measurements of lung and bone metastasis under the three treatment conditions. (**a**) MDA/Luc metastasis in the lung measured by luciferase activity (normalized by tissue weight). (**b**) Immunohistochemical (IHC) staining of human mitochondria in metastasis-affected lungs. (**c**) MDA/Luc metastasis in the hind-leg bone measured by luciferase activity (normalized by tissue weight). (**d**) IHC staining of human mitochondria in affected hind-leg bones (decalcified). Statistical comparisons are Student’s t-test. For all experiments, N = 5 animals per condition. Error bars: SEM. (**b**,**d**) large images are at 10× magnification; insets: 20× magnification image of the blue-arrow indicated area. Scale bars: 100 μm.

Metastasis accounts for over 90% of all cancer-caused deaths^19^. Extensive macrophage infiltration has been associated with poor patient prognosis and increased metastasis in many cancer types^20^,^21^. Luciferase activities in tissue lysates were measured as an indicator of metastasized MDA/Luc cells in lung and bone^18^, two common metastatic sites for breast cancer. Strikingly, we found that the Late TX group had higher metastatic signal than NTX and early TX groups in both lung (vs. NTX: p = 0.06; vs. early TX: p = 0.03, Student’s t-test, Fig. 5a) and bone (vs. NTX: p = 0.04; vs. early TX: p = 0.07, Student’s t-test, Fig. 5c). Those peripheral tissues detected with high luciferase signals were processed for immunohistochemical (IHC) analysis, and metastatic MDA/Luc cells were visually confirmed with human mitochondria-specific staining in both sites (Fig. 5b,d).

The iCasp9 construct has been successfully validated *in vivo* in animal models of mesenchymal stromal cell therapies^14^ and first-in-human clinical studies of adoptive cell therapy^22^. We applied the same construct to study the consequence of removing a stromal cell from TME, using CAFs as an example. Our suicide gene-engineered stromal cell model allowed for selective, efficient elimination of stromal cells from TME at desired time points. The chemical inducer used in the study has been proved safe in animals and ruled out of known side effects^14^,^15^,^23^. Apoptosis was the mechanism of choice for cell deletion because it is highly-regulated, occurs often in tumor beds, and has low immunogenicity. The insights generated with this approach is expected to be applicable in several immune-based TME-targeting strategies, including the chimeric antigen receptor (CAR) T cell therapy, where cell killing by cytotoxic T cells are presumably through apoptosis^24^. Although our study was designed to evaluate the effect of CAF elimination, it is important to consider the apoptosis of intratumoral cells itself (by our engineered approach or via therapeutic targeting) as a physiological trigger as well.

There have been inconsistent observations in the outcome of CAF elimination in preclinical and clinical studies. Immuno-targeting CAFs through FAP has shown promising results in several preclinical models^8^,^9^. However, similar approach in human cancer patients has not yielded clinical efficacy^11^,^12^. A preclinical study to eliminate FAP+ cells using chimeric antigen-receptor (CAR) cytotoxic T cells showed disruption of tumor desmoplasia^10^. Yet, two experimental models using conditional ablation^25^ and CAR T cell therapy^13^ to eliminate FAP+ cells both resulted in cachexia, an undesired off-tumor side effect. In addition, human stromal cells may carry unique pathophysiological roles in the TME compared to their mouse counterparts in the animal models. As xenograft animal models have more faithful representation of human tumor biology and better predicting efficacy on therapeutics than allograft models^26^, elimination of stromal cells of human origin in TME can provide unique insights in tumor biology, and potentially address the difference in the efficacies of CAF-targeting therapies under preclinical and clinical settings.

Our study revealed increases of lung and bone metastases with late elimination of human CAF cells in TME, which was associated with tumor-associated macrophages. Since CID was administered systemically through an intraperitoneal injection (not intratumoral injection), it is not likely the cause of a local macrophage response. Other *in vivo* studies have also concluded the safety of the CID drug^14^,^15^; the biological inertness and *in vivo* safety of a functional identical analog of the CID drug has been tested in a Phase I clinical trial^22^. The increased presence of macrophages can be a physiological response to the induced CAF apoptosis in tumors^27^. The role of macrophages in metastasis of various cancer types has been well documented^28^ and further studies will be needed to elucidate the precise mechanism causing the increased metastatic burden, with regard to the timing of the elimination and intratumor phenotypes. Early CAF elimination also increased the number of macrophages in the tumor sections compared to no treatment group, though the trend increased to a tipping point with a later elimination point. Isolation of these tumor associated macrophages to understand their differentiation status may be a next step to validate their tumor growth properties.

The scope of our study excludes the conclusion that CAFs prevent metastasis, though there are several insightful studies that have defined causal interactions between CAFs and cancer spread. Another potential mechanism of CAF-regulated metastases is associated with the heterotypic signaling between cancer cells and CAFs^3^,^4^,^6^,^29^,^30^,^31^,^32^. In triple-negative breast cancer, CAFs were shown to select for bone-specific metastatic traits in primary tumor cells, which thrive on CAF-derived factors CXCL12 and IGF1^33^. While 3 days vs. 10 days are unlikely to make a drastic CAF-selected clonal expansion of cancer cells in our model, the survival of the cancer cells with metastatic preference to bone and lung may have benefitted from the longer co-existence of CAFs in the macrophage-infiltrated tumor environment. Further time-lapse elimination studies will allow elucidation of the selection pressures imposed on cancer cells by TME, and the role cancer-CAF signaling in cancer cell survival.

The host response to tumor implantation served as a guide for our elimination time points. There was an interesting clustering of early and late stage cytokine responses that signaled a pro-inflammatory environment that was generated upon xenograft formation. The initial tumor burden was also reduced at this phase of tumor growth, with surviving cancer cells then reaching log phase proliferation in a matter of weeks thereafter. As CAFs may be responsive to each of these cytokine waves^16^,^34^, these cytokine clusters may collectively represent two different signals for CAFs to undergo differentiation and survival. Furthermore, the interaction between CAFs and innate immune cells such as macrophages in the tumor bed is complex and may have changing dynamics from a suppressive to a pro-growth state during the course of tumor progression^35^.

CAFs are an important controller of tumor growth, but are only one of a diverse population of stromal cells that surround a lesion^36^,^37^. Creating time-controlled knockouts of different human stromal cells in tandem can be a viable option to map the contribution of these stromal cells over the course of cancer progression. When combined with targeted therapeutic strategies, this model system can give critical insight into the stage at which stromal elimination or inhibition can improve outcomes.

## Materials and Methods

### Cells

Cancer-associated fibroblast (CAF) were provided by Orimo lab and previously described by Kojima *et al.*^16^ MDA-MB-231 expressing green fluorescent protein (GFP) was a generous gift from Weinberg lab^38^. MDA-MB-231 cells were engineered to express firefly luciferase (MDA/Luc) with a lentivirus containing luciferase reporter following a previous report^39^. All cells were expanded in Dulbecco’s Modified Eagle Medium (DMEM), 10% FBS, 100 U ml^−1^ penicillin, and 100 mg ml^−1^ streptomycin.

### CAF transformation

SFG.iCasp9.2A.ΔCD19 (iCasp) retrovirus were produced by transiently transfecting Phoenix Ampho cell line (National Gene Vector Biorepository, NGVB) with Lipofectamine 2000 (Life Technologies). Cells were rinsed after 24 hours, and collected for supernatants after additional 48 hours of incubation. Supernatant containing viral particles were mixed with regular DMEM full medium containing 8 μg/mL polybrene (Sigma), applied to CAF cell culture, and incubated overnight. The transformation were repeated three times. Cells were then immunostained and flow sorted for CD19 positive population.

### Antibodies and reagents

Antibodies used for staining included: anti-CD19 (clone SJ25C1, 1:50, flow cytometry), F4/80 (clone BM8, 1:100, immunohistochemistry) (eBioscience), and anti-mitochondria (clone 113-1, 1:100) (Millipore). Annexin V PE apoptosis detection kit was purchased from eBioscience. Luminex xMAP multiplex kits for mouse immune cytokines were purchased from Millipore. MTT cell proliferation assay kit was purchased from ATCC. All assays were run following the manufacturers’ instructions.

### MDA/Luc + CAF-iCasp xenograft tumor model

6-week old *NOD.CB17-Prkdcscid/J* female mice were purchased from Jackson Laboratory and maintained in the animal facility at MGH. Experiments were approved by and conducted in accordance with the policies of the Institutional Animal Care and Use Committee of MGH. MDA/Luc and CAF-iCasp cells were resuspended in phosphate buffered saline (PBS) and 1:1 mixed. The mixture was then further mixed 1:1 with high-concentration matrigel (BD Bioscience) and injected in the two abdominal mammary fat pads (1.6 × 10^6^ total cells per site in 100 μl volume). For each animal to be treated with CID drug, 5 μL of CID stock solution in ethanol (10 mg/mL) was diluted in saline and injected intraperitoneally (i.p.) at a dose of 50 μg per animal, and a second identical dose was administered 24 hours later. Tumors were allowed to growth for 3, 10, 15, and 30 days (for time lapse characterization) or 8 weeks. Tumor sizes was measured with caliper of the two perpendicular (longest/shortest) axes in the x/y plane, with tumor volume calculated as πxy^2^/6 assuming an ellipsoidal shape. For each endpoint, lung, liver, kidney, spleen and hind leg bone from sacrificed animals were snap-frozen in liquid nitrogen for *ex vivo* luciferase activity assay. Tumors were extracted and cut in halves, with half snap-frozen for cytokine multiplex assay and the other half fixed in 10% formalin, dehydrated in 70% ethanol, and processed for standard immunohistochemical analyses.

### Detection of metastases by *ex vivo* luciferase activity assay

Tissues of interest were individually pulverized into a fine powder by hand grinding with a dry ice-chilled porcelain mortar and pestle, and transferred to 1.5 ml tubes on dry ice. Grinded tissues were weighted and added with Promega Reporter Lysis Buffer, vortexed for 15 min, frozen and thawed three times with alternating liquid nitrogen and 37 ^o^C water bath, and centrifuged at 12,000 × g. 20 μl of each supernatant was mixed with 100 μl of Luciferase Assay Reagent (Promega) and measured for luminescence in a non-transparent white plate (Corning) by BioTek Synergy 2 plate reader. The luciferase activity in lysate was normalized to the measured tumor and tissue weight for statistical comparison.

### Immunohistochemistry

Fixed tumor samples were processed, embedded in paraffin, and sectioned in 5 μm thickness by Specialized Histopathology Services at MGH. Tissue sections were stained with antibodies against human mitochondria and mouse F4/80, respectively. Images were scanned by Nanozoomer 2.0RS (Hamamatsu Japan). For mitochondria stain, cells with distinct epithelial and fibroblastic morphology were counted per 20× field of view in tumors extracted on day 15. For F4/80 quantification, F4/80 density was calculated as the proportion of positive staining in each field of view at 5×.

### Statistical analysis

All data are presented in mean ± standard error of the mean (SEM), as stated in the figure legends. Statistical significance was assessed using Student’s *t*-test for pair-wise comparison, and 1-way ANOVA for comparison between multiple (≥3) conditions; p < 0.05 was considered as significant.

## Additional Information

**How to cite this article**: Shen, K. *et al.* Suicide Gene-Engineered Stromal Cells Reveal a Dynamic Regulation of Cancer Metastasis. *Sci. Rep.* **6**, 21239; doi: 10.1038/srep21239 (2016).
